# TeleHCV: A single-visit protocol and minimal passive remote monitoring are sufficient to achieve high SVR with a sofosbuvir-velpatasvir regimen

**DOI:** 10.1016/j.clinsp.2025.100643

**Published:** 2025-04-23

**Authors:** Jerônimo De Conto Oliveira, Fernando Comunello Schacher, Marisa Boff Costa, Maurício Godinho Kolling, Raquel Boff Costa, Henrique Cabral Scherer, Paula Martins Fernandes, Natan Katz, Marcelo Rodrigues Gonçalves, Dimitris Varvaki Rados, Mário Reis Álvares-da-Silva

**Affiliations:** a Graduate Program in Gastroenterology and Hepatology, Universidade Federal do Rio Grande do Sul, Porto Alegre, RS, Brazil; b GI/Liver Unit, Hospital de Clínicas de Porto Alegre, Porto Alegre, RS, Brazil; c Núcleo de Telessaúde Técnico Científico do Rio Grande do Sul (TelessaúdeRS-UFRGS), Porto Alegre, RS, Brazil; d Project ECHO Liver Diseases Clinic, HCPA, Porto Alegre, Brazil; e Universidade Federal do Rio Grande do Sul, Faculdade de Medicina, Porto Alegre, RS, Brazil; f World Gastroenterology Organisation Porto Alegre Hepatology Training Center, Porto Alegre, RS, Brazil; g Researcher, CNPq, Brazil

**Keywords:** Viral hepatitis, Hepatitis C, Treatment, Direct-acting antivirals, Minimal monitoring, Telemedicine, Telehealth, Microelimination, Public health, Care cascade

## Abstract

**Aim:**

Chronic Hepatitis-C Virus (HCV) treatment has evolved significantly in recent years with Direct-Acting Antivirals (DAAs). The traditional care cascade includes several steps that limit its impact, and simplification protocols have emerged. Therefore, the authors explore a simplified treatment strategy for HCV in a healthcare system with limited access to specialized care.

**Subject and methods:**

Chronic HCV, DAA-naïve patients waiting for in-person specialized care were invited to a single-arm non-inferiority trial to evaluate a simplified treatment protocol with a single face-to-face appointment and minimal monitoring of antiviral therapy. The unique visit consisted of an HCV presentation followed by individual medical consultation, blood tests, and delivery of sofosbuvir-velpatasvir pills for a 12-week treatment. Patients were remotely monitored without scheduled on-treatment appointments or phone calls. After treatment, teleconsultation using video was offered. The primary outcome was Sustained Virological Response (SVR) 12-weeks post-treatment. It was analyzed with Intention-To-Treat (ITT) and Per-Protocol (PP) approaches. ClinicalTrials.gov: NCT04039698.

**Results:**

The authors included 144 patients, of which 54.2 % were male, mean age was 52 years. Most individuals (84.7 %) had an APRI score < 1. All patients received at least one dose of DAA, 139 completed antiviral therapy, and 131 had SVR evaluation. The ITT SVR rate was 90.3 % (130/144 patients; 95 % CI 84.2 %‒94.6 %), and the PP SVR was 99.2 % (130/131 patients; 95 % CI 95.8 %‒100 %). Eighty-three adverse events were reported, and 93 % were handled with remote care.

**Conclusion:**

This simplified strategy achieved a high SVR rate in a population with restricted access to specialized care. Telehealth tools and minimal monitoring are promising components for policies aimed at HCV elimination.

## Highlights

• Direct-acting antivirals are highly efficacious for hepatitis C virus cure.
• Access to specialized care and antivirals are difficult in some areas.
• Simplifying delivery and follow-up of therapy can facilitate hepatitis C cure.
• A single-day visit protocol achieved acceptable cure rates in this study.
• This strategy.

## Introduction

Hepatitis-C Virus (HCV) is a significant public health issue, with >50 million people estimated to be chronically infected globally,^1^ one of the leading causes of cirrhosis and Hepatocellular Carcinoma (HCC). In the last decade, its treatment has been revolutionized by the highly efficacious and well-tolerated Direct-Acting Antivirals (DAAs),^2^ enabling cure in almost every patient. This simple and efficient therapy led to the World Health Organization's (WHO) establishing the goal of eliminating HCV globally by 2030.^3^ However, few countries are currently on the route to HCV elimination,^4^^,^^5^ stressing that the authors still need to overcome barriers to achieve this objective, especially outside high-income countries.

Previous data have reported an overall 97 % Sustained Virological Response (SVR) in different groups globally and in Brazil. Although over 12 million have an HCV infection diagnosed by the end of 2020, only 641,000 were estimated to have begun treatment in 2020.^1^ Furthermore, international data have shown that up to 70 % of diagnosed patients do not complete all the steps of the care cascade toward cure.^6^ These data emphasize the need to simplify access to DAAs and the whole care cascade and simplified treatment protocols have been published^7^ and included in international guidelines.^8^ In Brazil, the public health system provides universal, free-of-charge DAA treatment.^9^ Despite this effort and investment, Brazil is not on route to eliminating HCV by 2030.^4^^,^^5^

Many HCV macro and micro-elimination strategies have been created around the world,^10^^,^^11^ and telehealth has been reported to aid in HCV treatment in several settings.^12^^,^^13^ Telemedicine tools are powerful in connecting patients to specialized care^14^ and have expanded their boundaries exponentially after the COVID-19 pandemic.^15^ Despite high heterogeneity in usage, remote care is part of health care delivery.^16^^,^^17^ Brazil, which has an estimated 0.7 % prevalence of chronic hepatitis-C,^9^ is a middle-income, continental-sized country suitable for telehealth applications. The authors developed a single-arm clinical trial to evaluate if a simplified HCV treatment protocol leads to a rate of SVR similar to the results from medical literature.

## Methods

### Study design and participants

This is an open-label, single-center, single-arm clinical trial, joining an academic university hospital (Hospital de Clínicas of Porto Alegre) with a primary care-oriented telehealth research program (TelessaudeRS-UFRGS). The current report follows the CONSORT Statement.^18^ It is registered at ClinicalTrials.gov, NCT04039698, and the Brazilian platform of clinical studies, number CAAE 91,278,418,200,005,327, in which full protocol is available. Selected patients could live in any of the 497 cities of Rio Grande do Sul, the southernmost Brazilian state.

Patients on the waiting list for consultation with a gastroenterologist or infectious disease physician at the web-based Rio Grande do Sul state's platform for ambulatory referrals (GERCON; https://gercon.procempa.com.br) were screened using the International Classification of Diseases (ICD-10) codes for chronic hepatitis-C (B18.2) and acute hepatitis-C (B17.1).

The eligibility criteria were: 1) At least 18-years of age and 2) Chronic HCV-confirmed viremia. Exclusion criteria were high suspicion or confirmed diagnosis of cirrhosis, defined by 1) Liver stiffness ≥12.5 kPa on transient elastography, 2) METAVIR score of F4 in liver biopsy, 3) Clinical, ultrasound, or endoscopic evidence of cirrhosis or portal hypertension, 4) Platelets < 150.000 mL, and 5) AST-to-platelets Ratio Index (APRI) ≥ 2.0 or FIB-4 score > 3.25. Patients who experienced DAAs, were pregnant or breastfeeding, had a glomerular filtration rate of <30 mL/min, had previous solid organ transplantation, or would be exposed to major drug-drug interactions with sofosbuvir-velpatasvir were also excluded. Severe comorbidities also lead to exclusion, as treatment without additional in-person visits was considered risky by the investigators.

This study received approval from the Ethics Committee of Hospital de Clínicas de Porto Alegre (CAAE 91,278,418,200,005,327). All patients provided written informed consent.

### Study procedures

Patients meeting the inclusion criteria were contacted by phone by a research assistant to be invited to the study and receive explanations of the research procedures. After verbal consent, the authors verified personal information and collected data on mobile phones and internet access. Finally, the authors scheduled an in-person visit at the Clinical Research Center of Hospital de Clínicas de Porto Alegre. If not previously available on the GERCON platform, patients were asked to send HCV-related test results to the research team through an instant messaging application (WhatsApp®) before the visit. The study physician received these results, uploaded them to the RedCap® platform, and finally deleted them from WhatsApp® and smartphone registries. In addition, all patients were asked to bring recent (i.e., from the previous 12-months) blood test results to the visit. During the SARS-CoV-2 pandemic, researchers used instant messaging to screen for symptoms of COVID-19 on the day before the visit.

**Fig. 1:**
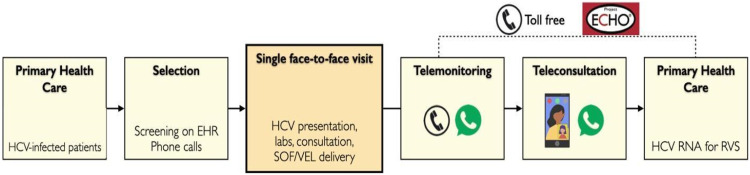
Steps of study procedures. HER, Electronic Health Records; SVR, Sustained Virologic Response; SOF/VEL, Sofosbuvir and Velpatasvir.

Fig. 1 illustrates the steps of study events. On the day of the meeting, patients spent up to four hours in a fast-track evaluation protocol which consisted of a group activity (group presentation and question-and-answer session), medical and pharmacist consultation, collection of blood tests, and delivery of antivirals.

Patients initially joined a group activity in a small conference room with up to 13 patients (limited to 6 persons during the COVID-19 pandemic), comprising a 5 min educational at distance HCV lecture through Project ECHO™ platform (Project ECHO Liver Diseases Clinic, HCPA, Porto Alegre, Brazil), followed by detailed explanations on the study protocol and the question-and-answer session to solve patients’ doubts.

After privately giving signed informed consent, patients had a focused medical consultation. HCV status, comorbidities, medications, and previous laboratory tests were analyzed, and inclusion and exclusion criteria were reviewed. Those included in the study received a prescription of the daily co-formulated tablet of sofosbuvir 400 mg and velpatasvir 100 mg for 12 weeks. In addition, they were given a requisition form to collect HCV RNA 12 weeks after the end of the treatment in their local laboratory. The project physician checked drug interaction through the University of Liverpool website^19^ and made prescription adjustments when necessary. Immediately after the visit, patients collected blood tests (complete blood count, aspartate aminotransferase, alanine aminotransferase, and creatinine). Following HCV Brazilian guidelines, HCV RNA was collected in patients without this test in the previous 12-months.^9^ HCV RNA concentrations were quantified using the Abbott RealTime HCV test (Abbott, Chicago, IL, USA; lower limit of quantification 12 IU/mL).

After these steps, every patient had an individual pharmacist consult to provide orientations on administering the tablets. Three packages of antiviral with 28 tablets each were dispensed, covering the entire 12-week course. Next, patients were asked to start treatment, taking the first tablet of antivirals in front of the pharmacist. From this point, the remaining study procedures were performed by distance: monitoring of adverse events, collecting HCV RNA after 12 weeks of the end of antiviral treatment, and a final telehealth appointment by a video call.

During the treatment period, there were no additional visits or programmed contact. Researchers presented the study's procedures to patients, focusing on teleconsultation after the end of antiviral treatment. The Primary Care Physicians (PCP) also had access to a toll-free number for telephone consultations with specialists and generalists of TelessaúdeRS-UFRGS.^20^ In addition, patients received written information on antiviral treatment, treatment sequence (i.e., end of treatment expected date and when to collect HCV RNA), and how their PCP could contact TelessaudeRS or join Project ECHO^TM^ sessions. The authors encouraged the patients to show this information PCP or primary care nurse. Besides, the authors indicated that patients should search for local and in-person evaluation whenever necessary, reinforcing that the research team could perform orientations and consultations by distance.

For telemonitoring purposes, all patients were instructed to contact the research team during treatment using mobile phone instant text messages or phone calls. A phone or video call could be scheduled or performed instantly at the physician's discretion responsible for that evaluation.

A text message was sent to each patient at the end of the treatment period to schedule a final video call and remind them to collect the HCV RNA after a 12-week interval.

HCV RNA was collected locally, and all patients were asked to send the test result to the research team. The research physician also had access to the Brazilian official system for laboratory results (https://gal.riograndedosul.sus.gov.br), in which molecular biology test results are reported. Patients were considered lost to follow-up if unreachable by multiple phone calls or text messages after >18 months of the end of the treatment.

### Outcomes

The primary outcome was Sustained Virological Response (SVR) at least 12 weeks after the end of the treatment, evidenced by HCV RNA concentration below the limit of detection. The authors performed a non-inferiority comparison with the 97 % SVR rate achieved by conventional face-to-face treatment in national and international real-life studies.^21^, ^22^, ^23^

The incidence of Adverse Events (AE), Serious Adverse Events (SAE), and adherence to the study medication by self-reported grading using a 0 to 10 scale were evaluated as secondary outcomes. Adverse effects were graded by the Division of Acquired Immune Deficiency Syndrome Table from the National Institutes of Health (version 2.0).^24^ Adverse events reported between the first dose and twelve weeks after the end of treatment were considered potentially related to the antivirals.

The authors also collected social, educational, and income data to assess potential limitations to telemedicine in some socioeconomic settings. Finally, to evaluate adherence, patients were asked to grade themselves from 0 to 10 regarding compliance with study medications and to quantify the frequency of missed tablets.

### Statistical analysis

The primary (intention-to-treat) analysis included all patients who received at least one tablet of the study medication. Patients who performed the final HCV RNA assessment comprised the per-protocol population. Missing data on clinical variables were considered absent and not included in the statistical analysis for that determined variable.

One sample binomial test (Clopper-Pearson exact test) was used to compare the SVR rate of the sample with the hypothesized 97 % parameter of the historical cohorts. The chi-Square test was utilized for comparing factors associated with SVR.

Study data were collected and managed using Research Electronic Data Capture (REDCap) tools hosted at Hospital de Clínicas de Porto Alegre.^25^^,^^26^ Statistical analysis was performed in IBM SPSS® (version 23). Primary Care Assessment Tool (PCATool)^27^ was used to investigate patients' access to health care.

#### Sample size estimation

Sample size calculation assumed a 97 % efficacy for SVR for both groups (historical data and experimental group). The authors established the non-inferiority limit at 5 % and a one-sided 95 % Confidence Interval (α = 0.05). It was estimated that 144 patients would be required to achieve a power of 80 % to affirm that there was no significant difference in SVR rates between experimental treatment and historical data.

## Results

**Fig. 2:**
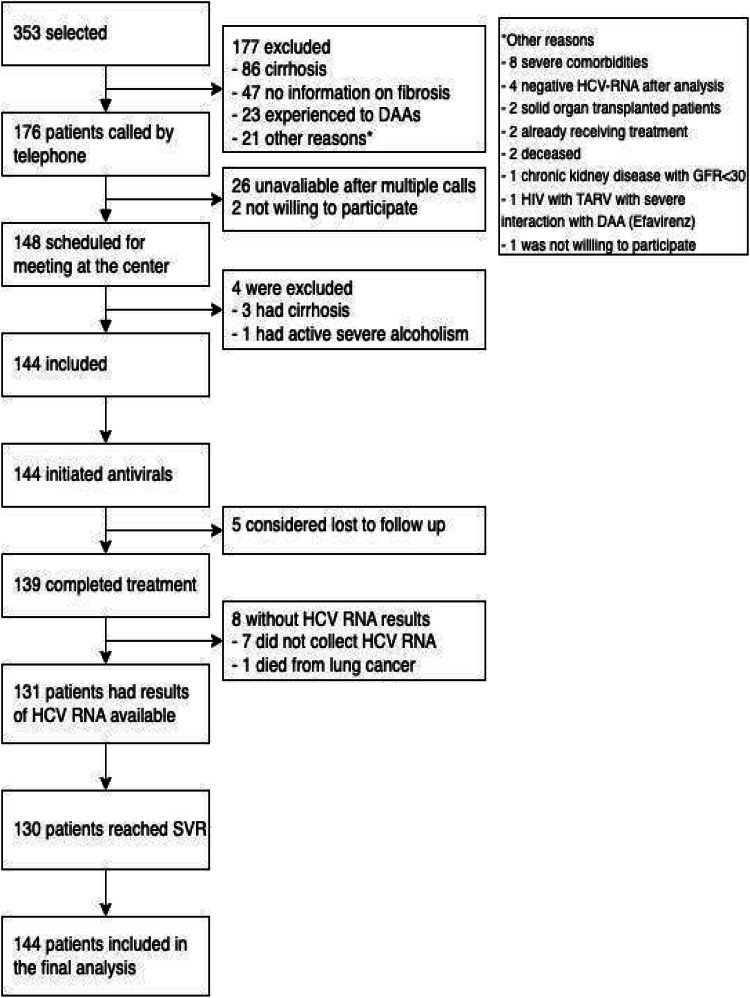
Flowchart of study participants.

The flowchart of patients is shown in Fig. 2. Enrollment started on August 8, 2019, and ended on December 10, 2020. The authors screened 353 subjects with inclusion criteria and identified 176 patients without exclusion criteria. The most frequent reason for exclusion was the presence of cirrhosis (86 patients), followed by 47 with insufficient information on fibrosis staging, 23 having previous DAA treatment, and 21 exhibiting other exclusion criteria. The authors made phone calls to 176 patients and reached 150 patients. Two patients refused to participate in a research protocol alleging they were already about to initiate antiviral treatment. Finally, 148 patients had a visit scheduled at the research clinic, and 144 patients were enrolled (4 additional patients were excluded – detailed in Fig. 2).

Table 1 presents the baseline characteristics of the enrolled patients. Most patients were male, and the mean age was 52 years. APRI score inferior to 1.0 was present in 84.7 % of patients. Eight patients did not have quantitative assay HCV RNA results available for the consultation but had a recent genotyping result.

**Table 1:** Demographic and baseline characteristics of enrolled patients (*n* = 144).

**Sex**	
Female	66 (45.8 %)
Male	78 (54.2 %)
**Age**	
Mean (SD)	52 (12.9)
Range	20‒82
**HCV genotype**	
1a	32 (22.2 %)
1b	15 (10.4 %)
2	10 (7.0 %)
3	33 (22.9 %)
Missing	54 (37.5 %)
**Laboratory results ‒ median (IQR)**	
AST	45 (28)
ALT	58 (65)
Platelets	226 (77)
**Fibrosis staging**	
APRI ‒ median (IQR)	0.49 (0.42)
< 0.5	73 (50.7 %)
0.5‒0.99	49 (34.0 %)
1.0‒1.49	7 (4.9 %)
1.5‒2.0	7 (4.9 %)
> 2.0	8 (5.5 %)
**Metavir score (biopsy or TE)**	
F0/F1	19 (13.2 %)
F2	10 (7.0 %)
F3	2 (1.4 %)
F4	1 (0.7 %)
Unavailable	112 (77.7 %)
**Common comorbidities**	
Hypertension	53 (36.8 %)
Diabetes mellitus	20 (13.9 %)
HIV	1 (0.7 %)
**Reported current/former drug use**	
Cannabis	7 (4.9 %) / 29 (20.1 %)
Inhaled cocaine	3 (2.1 %) / 20 (13.9 %)
Intravenous cocaine	0 (0 %) / 11 (7.6 %)

Data are n ( %) unless otherwise stated.

APRI, AST-to-Platelets Ratio Index; TE, Transient Elastography.

All 144 patients received the first sofosbuvir-velpatasvir tablet and 139 completed antiviral treatment. From them, 131 collected blood samples for SVR assessment. Seven patients reported complete use of antivirals but did not collect HCV RNA until the current analysis; at last, one patient died from lung cancer before collecting HCV RNA for SVR. Five patients could not be reached by telephone or text messages and were considered lost to follow-up. The socioeconomic aspects of study participants are summarized in Table 2: most patients were poor and had less than five years of education. Before this study, only six participants had ever gotten in touch with a physician remotely (by phone, text messages, or teleconsultation).

**Table 2:** Sociodemographic characteristics of study participants.

**Monthly family income (*n*=136)**	
<2 minimum Brazilian wages^a^	79 (58,1 %)
>2 minimum Brazilian wages^a^	50 (36,8 %)
Did not provide information	7 (5,1 %)
**Years of study**	
5-years or less	85 (62.5 %)
>5-years	51 (37.5 %)
**Area of residence**	
Rural	19 (14.0 %)
Urban	117 (86.0 %)
**Health access (*n*=87)**	
First access through the primary care unit	75 (86.2 %)
Reported difficulties in obtaining a medical consultation in a primary care unit	38 (43.7 %)

Data are n ( %) unless otherwise stated.

a Two minimum wages in Brazil mean an amount of BRL 2385,00 in 2021.

**Fig. 3:**
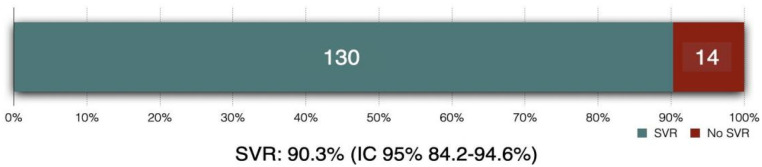
Intention-to-treat sustained virological response (SVR).

**Fig. 4:**
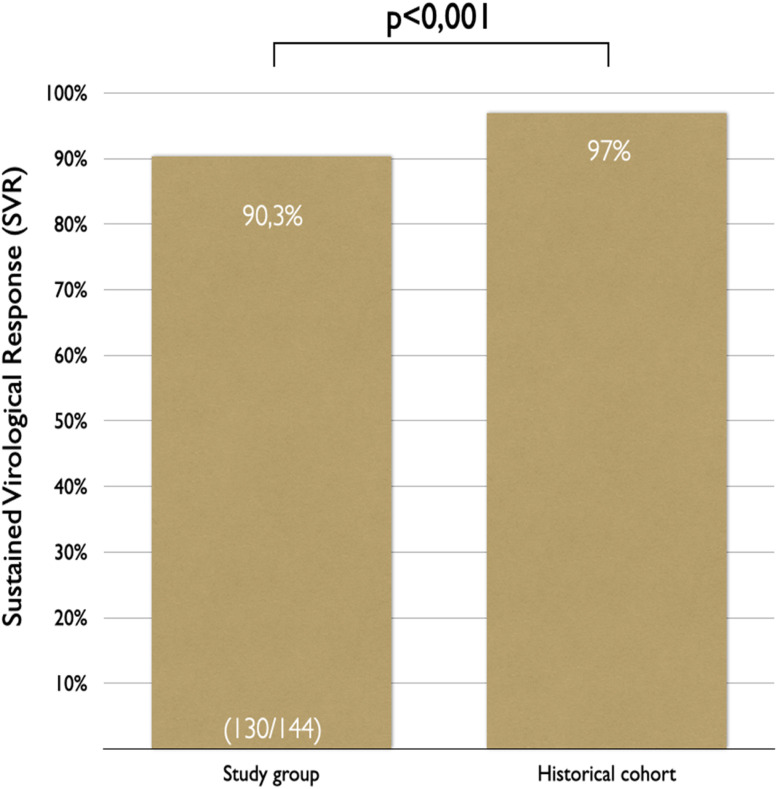
Intention-to-treat comparison between the study sample and historical cohort.

In the intention-to-treat analysis, 90.3 % (95 % CI 84.2 %‒94.6 %) of patients achieved SVR (Fig. 3, Fig. 4). Clopper-Pearson exact test demonstrated that the SVR obtained in this group was statistically inferior (*p* < 0.001) to the historical results, rejecting the non-inferiority hypothesis. Per protocol analysis, the SVR rate was 99.2 % (130/131 patients; 95 % CI 95.8 %‒100 %); for this analysis the non-inferiority margin was not touched, and inferiority was discarded.

Only one patient had a positive viral load after the end of treatment. This unique virologic failure occurred in a genotype three infected patient who reported good adherence to antivirals and had a platelets count over 200.000 mL at the screening. Eight patients without diagnosed cirrhosis presented thrombocytopenia or APRI scores above 2.0 on laboratory tests performed at the face-to-face meeting and were maintained in treatment protocol and included in the final analysis. Seven patients completed treatment and achieved SVR, while one did not collect HCV RNA after treatment.

There was no association between not having collected HCV RNA after treatment and the following factors assessed: social, demographic, or clinical characteristics of patients, current or previous use of illicit drugs, or history of alcohol abuse.

Only six patients completed the post-treatment 12 weeks before the SARS-CoV-2 pandemic restrictions took place in Brazil.

HCV RNA reports were actively sent by 60 patients (45.8 %), upon request by 24 subjects (18.3 %), and 47 patients (35.9 %) received their HCV RNA report from the research.

A total of 83 AEs were reported by 54 patients. Six patients required an unscheduled health care meeting: four had a primary care consultation, one sought the dentist, and one went to the emergency department due to urinary retention. The remaining adverse events were managed by text messages (69 times) or video or phone calls (7 times). Three patients had serious adverse events, none considered related to the study procedures or antiviral treatment. One patient with tooth pain needed a dental procedure; one needed to visit an emergency department; one death was reported during follow-up (due to lung cancer). Not a single PCP joined a Project ECHO^TM^ session.

Good adherence to antiviral treatment was reported for those with collected data (123 subjects ‒ 91.9 %). Only ten subjects (8.1 %) forgot a pill more frequently than once a month. Eighty-seven patients answered questions on public health access: 75 (86.2 %) usually sought the primary care unit in the first place when a routine medical appointment is needed. Difficulties in obtaining a face-to-face medical consultation were referred by 38 subjects (43.7 %), the same number that reported not having access to orientations by a phone call from a primary care health professional.

## Discussion

In this non-randomized clinical trial, the authors reported the first experience in HCV treatment in Brazil using minimal monitoring with telemedicine tools. By offering simplified access to DAAs through care coordination with a multidisciplinary team, on-site distribution of DAAs, telemedicine follow-up, and patient education, the authors achieved an SVR rate higher than 90 % in a population with restricted access to specialized care. Nevertheless, this difference was not non-inferior to the historical benchmark.

The authors attained a higher proportion of patients with proper assessment (144/176), treatment initiation (144/144), and completion (139/144) than national and international historical cohorts with similar populations.^6^^,^^21^^,^^22^^,^^28^ The elevated proportion of eligible patients in the study brings to light its pragmatic profile. The 90.3 % SVR rate achieved was inferior to the 97 % from historical data and real-life studies,^21^^,^^22^ albeit it surpasses the WHO parameter of 85 % reported in the 2015.^29^

On the other hand, in a per-protocol analysis that included patients who had final HCV RNA data, the SVR rate was 99.2 % (130/131 patients). In this analysis, non-inferiority was demonstrated. The single patient without SVR had unidentified cirrhosis and was subsequentially diagnosed with an HCC, a condition associated with lower SVR rates in this genotype.

To our knowledge, the present study uses the most straightforward monitoring protocol with the least frequent contact with patients during antiviral treatment. Previous similar studies had a more active assessment^30^ or monitored patients by regular messages or phone calls.^31^

High SVR rates were reported in previous studies in Brazil without telemedicine. A nationwide, open-label, single-arm trial achieved an SVR rate of 96.4 % in patients with advanced fibrosis and genotype 1 infection.^22^ Three real-world data studies had reported similar efficacy: a national study with 3939 patients achieved SVR rates over 95 %,^32^ while one performed in southern Brazil achieved a 95 % SVR rate with a range of DAA-containing regimens,^33^while in our center, DAA treatment patients cured 95.5 % of HCV-infected patients after liver transplantation.^23^ When compared to this report, cited studies had a much higher proportion of interferon-experimented patients (> 50 % vs. 0.7 %) and had major industry-derived or private clinic-related care. None of them reported the socio-economic characteristics of the included subjects, preventing this comparison. Regarding the failure to achieve non-inferiority, patients of the trial (public, primary-care derived, with poor socioeconomic status) are prone to limited access to health, including viral load testing, which could at least partially explain our lower SVR rate.

Previous studies successfully approached HCV treatment with different telemedicine tools, such as telemonitoring,^31^ teleconsultation,^12^^,^^34^^,^^35^ and telementoring,^36^ achieving SVR rates comparable with those in conventional treatment groups. Simplified monitoring was also explored in a randomized clinical trial with 380 patients treated with eight weeks of glecaprevir and pibrentasvir, with a high SVR rate on the simplified monitoring arm, although not equivalent to the standard monitoring schedule group (92 % × 95 %).^31^ Brazil has a long history of telemedicine usage^37^: teleconsultation, regulation, and diagnosis services are available, focusing on primary care.^14^^,^^38^^,^^39^ Despite this, the authors are unaware of similar strategies for HCV in Brazil.

A multicenter, open-label, single-arm trial with sofosbuvir and velpatasvir with minimal monitoring reported a 95 % SVR rate.^7^ Patients had a single visit to the research center and no additional scheduled appointments, while two remote contacts were programmed at 4 weeks and 22 weeks after antiviral initiation. The present study achieved lower results and had no regular contact programmed for monitoring purposes. An additional but significant difference is that, in the cited study, patients collected blood samples for HCV RNA after treatment in the same research center where they were initially treated. In this pragmatic study, the authors provided treatment with simplified access to an underserved, vulnerable population, positively impacting the cascade of treatment.^6^^,^^28^ Social vulnerability might have imposed difficulties for patients to access local services in the studied population. As mentioned, most patients had incomplete primary education, and 61.2 % had monthly earnings less than two national minimum wages for their families. Nevertheless, the subjects had previously HCV-RNA confirmed infection, possibly excluding even more vulnerable patients. Patients who did not collect HCV RNA for SVR analysis accounted for most treatment failures in our protocol. Project ECHO found a similar problem when scrutinizing the cascade of care from 100 patients lacking proper healthcare access.^36^ There were no significant differences in clinical or socioeconomic aspects between patients who either haven't collected HCV-RNA or have been lost to follow-up after treatment and those who did. Nonetheless, the study wasn't powered for this comparison, therefore attrition bias cannot be excluded.

PCPs in charge of the study patients did not join the offered Project ECHO^TM^ sessions and performed fewer teleconsultations than the authors anticipated. This demonstrates a lack of interest and engagement in HCV treatment, a considerable problem in HCV elimination in Brazil, whose public health system is based on Primary Healthcare (PHC). In addition, the COVID-19 pandemic might have restrained PCPs' interest in HCV treatment during this period.

COVID-19 had unprecedented effects on the global economy and had a huge impact in health access in Brazil. Social isolation driven by the pandemic took place during the recruitment period, further restricting several regular services, including public transportation, elective medical appointments, and blood sample collection, with negative impacts on our study as well. On the other hand, the usage of telehealth has grown exponentially at that time, which may have contributed to patient confidence in the telemedicine protocol of our study. As mentioned before, only six patients completed the post-treatment 12 weeks in pre-pandemic era.

Despite not achieving the non-inferiority margin, this study has its strengths. First, the authors have shown that remote HCV treatment is feasible in the Brazilian public healthcare system. Second, SVR rates in the per-protocol analysis were comparable to the historical benchmark.

This study also has several limitations. The open-label, single-arm design has intrinsic limitations on causality. Although a randomized controlled trial would have generated more robust results, several studies on HCV treatment in recent years were single-arm trials, including those on minimal monitoring.^7^^,^^40^ Besides, the authors had a major concern about the ethical appropriateness of an RCT in this pragmatic study: at that time in Brazil, patients had to wait several months to access antivirals on the “standard treatment”. Additional specific limitations must be mentioned. First, the minimal monitoring strategy may have been detrimental to the results since almost 10 % of patients did not collect SVR testing after treatment. Second, the unsuccess in engaging PCPs in caring for these patients added frailty to our follow-up strategy. Third, regarding the care cascade analysis, it should be noticed that our patients have already engaged on their route to treatment once the authors selected patients with HCV-RNA confirmed infection waiting for specialized care. Finally, the authors analyzed adherence by self-report and only for those who completed the treatment protocol.

Even though SVR evaluation is the standard practice worldwide, a “treat and forget” strategy without SVR assessment may be considered soon; at least in underserved populations, treating more patients more straightforwardly is preferable to no treatment. In the sample of vulnerable patients with restrained access to health services, most subjects without SVR had no HCV RNA test after treatment. Still, the SARS-CoV-2 pandemic might have played a role in the difficulties faced by patients in reaching local laboratories to perform HCV RNA tests: <5 % of them were able to perform SVR testing before social distancing restrictions imposed by mid-March of 2020, which had a tremendous impact in public services in Brazil.

Brazil is a continental-size country and has huge disparities. The present study population seems to be representative of the one that uses the public health system in Brazil, at least the one of the southern regions of the country. Study subjects were mainly poor, with low education, and dependent on the public health system and primary care. Nevertheless, this protocol might face additional difficulties in even poorer and vulnerable backgrounds like in some regions in Brazil. On the other hand, the authors meticulously planned and described the steps of this protocol, allowing it to be improved and refined, and maybe be replicated in other settings after adjustments for local needs.

In conclusion, although not reaching the primary non-inferiority outcome, this study showed that it is possible to treat hepatitis C remotely in Brazil, with an SVR higher than 90 %. This experience can help design public policies to eliminate hepatitis-C.

## Data availability

The submitted work should be original and should not have been published elsewhere in any form or language.

## Ethical approval

All procedures involving human participants were in accordance with the ethical standards of the Hospital de Clínicas de Porto Alegre's research committee and with the 1964 Helsinki Declaration and its later amendments or comparable ethical standards.

## Informed consent

All patients provided written informed consent.

## Funding, data management, and role of the funding source

The study was designed and performed exclusively by the investigators. The Brazilian Ministry of Health provided all the antivirals used in the study. The funders did not participate in any study design, selection of patients, interpretation of data, or article review.

## Funding

Brazilian Ministry of Health provided all antivirals used in the study; Fundo de Incentivo à Pesquisa (FIPE) ‒ Hospital de Clínicas de Porto Alegre provided funding for operational and laboratory costs; and Coordenação de Aperfeiçoamento de Pessoal de Nível Superior – Brasil (CAPES).

## Conflicts of interest

The authors declare no conflicts of interest.
